# The RNF26/CBX7 axis modulates the TNF pathway to promote cell proliferation and regulate sensitivity to TKIs in ccRCC

**DOI:** 10.7150/ijbs.69325

**Published:** 2022-02-28

**Authors:** Wentao Liu, Haohui Wang, Chengzhu Jian, Wei Li, Kun Ye, Jiannan Ren, Liang Zhu, Yinhuai Wang, Xin Jin, Lu Yi

**Affiliations:** 1 Department of Urology, The Second Xiangya Hospital, Central South University, Changsha, Hunan, 410011, China; 2 Uro-Oncology Institute of Central South University, Changsha, Hunan, 410011, China; 3 Hunan Engineering Research Center of Smart and Precise Medicine, Changsha, Hunan, 410011, China

**Keywords:** CBX7, RNF26, TNF signaling pathway, clear cell renal cell carcinoma

## Abstract

Clear cell renal cell carcinoma (ccRCC) accounts for 85% of all malignant renal tumors. Currently, the pathogenesis of ccRCC is not fully understood. Chromobox (CBX) family proteins are the major subunits of PcG complexes and are implicated in regulating mammalian development. The CBX family consists of eight members, namely, CBX1-8. Numerous studies have highlighted that each CBX protein exhibits distinct functions and prognostic roles in specific cancer types. In this study, in silico analysis indicated that CBX7 was downregulated in ccRCC and correlated with favorable prognosis in a ccRCC cohort. Subsequent studies showed that CBX7 inhibited cancer cell proliferation and invasion. Then, we showed that CBX7 downregulated ETS1 to inactivate the tumor necrosis factor (TNF) signaling pathway, which inhibited tumor proliferation and enhanced the sensitivity of ccRCC cells to tyrosine kinase inhibitors (TKIs). Moreover, we found that CBX7 was a bona fide substrate of RNF26. RNF26 promoted the degradation of CBX7 and enhanced ccRCC tumor growth. Therefore, our results revealed a novel RNF26/CBX7 axis that modulates the TNF signaling pathway in ccRCC.

## Introduction

Renal cell carcinoma (RCC) is a heterogeneous group of cancers derived from the renal epithelium 1. Clear cell RCC (ccRCC) is the major histological subtype of RCC and exhibits distinct characteristics, including genomic alterations and altered tumor metabolism 2. Approximately 85% of all RCC tumors are ccRCC tumors. Since ccRCC does not respond to traditional chemotherapy and radiotherapy 2, exploring novel therapeutic strategies has always been a hot spot in ccRCC research. Over the past two decades, targeted antiangiogenesis therapies, including tyrosine kinase inhibitors (TKIs), have profoundly increased the survival time of patients, due to the abnormal hyperactivation of the vascular endothelial growth factor (VEGF) pathway commonly observed in ccRCC 3, which implies that understanding the pathogenesis of ccRCC forms the basis for attempts to identify novel treatment options 4.

Polycomb group (PcG) complexes act as epigenetic regulators to repress target gene expression in cells 5. Chromobox (CBX) family proteins are the major subunits of PcG complexes and are implicated in regulating mammalian development 6. The CBX family consists of eight members, namely, CBX1-8. Numerous studies have highlighted that each CBX protein exhibits distinct functions and prognostic roles in specific cancer types 7. It has been mentioned that CBX4 is upregulated in ccRCC and associated with its unfavorable prognosis 8. Further exploration showed that CBX4 forms a complex with histone deacetylase 1 (HDAC1) to repress the expression of Kruppel like factor 6 (KLF6), which promotes cell growth and migration in ccRCC 8. However, the specific role of the remaining CBX family members in ccRCC remains elusive.

In this study, in silico analysis indicated that CBX7 was downregulated in ccRCC and correlated with favorable prognosis in a ccRCC cohort. Subsequent studies showed that CBX7 inhibited cancer cell proliferation and invasion. Then, RNA-seq data for CBX7 indicated that CBX7 inactivated the TNF pathway to block tumor growth and downregulated IL6 to sensitize renal cancer cells to TKIs. Moreover, we found that RNF26 functioned as an E3 ligase for CBX7 and promoted CBX7 degradation in renal cancer cells. Therefore, we identified an RNF26/CBX7 axis that modulates the TNF pathway to regulate the malignant biological behavior of ccRCC cells.

## Method and Materials

### Cell lines and cell culture

The two ccRCC cell lines that we cultured, 786-O and A498, were obtained from Yuchi Biology (Shanghai, China), and both cell lines were identified by short tandem repeat (STR) profiling. Cells were cultured in RPMI-1640 medium (Gibco, USA) or minimum essential medium (MEM, Gibco, USA) supplemented with 10% fetal bovine serum (FBS; AC03L055, Shanghai Life-iLab Biotech, China) and 1% penicillin, placed in an incubator at 37 °C in 5% CO2, and the culture medium was changed twice a week.

### Antibodies and chemical agents

The antibodies used for western blot analysis are as follows: GAPDH (#60004-1-Ig, Proteintech, 1:5000 dilution), RNF26 (#16802-1-AP, Proteintech, 1:800 dilution), IL6 (#21865-1-AP, Proteintech, 1:100 dilution), ETS1 (#12118-1-AP, Proteintech, 1:1000 dilution) and CBX7 (#26278-1-AP, Proteintech, 1:1000 dilution). NeutraKine® IL6 Monoclonal antibody (Cat no: 69001-1-Ig) 100ng/ml was obtained from Proteintech. MG132 (#S2619), IKK-16 (#S2882), JSH-23 (#S7351) and cycloheximide (CHX) (#S7418) were purchased from Selleckchem.

### Coimmunoprecipitation and western blot analysis

For coimmunoprecipitation (IP), 1 ml of RIPA buffer (#P0013, Beyotime, China) containing a protease inhibitor was added to a 10 cm dish of ccRCC cells for lysis on ice for 30 min. Then, the cell lysates were pipetted into a 1.5 ml Eppendorf tube and centrifuged at 12000 × g for 15 min. The supernatant was aspirated into a new 1.5 ml Eppendorf tube and incubated with protein A+G beads (#P2029, Beyotime, China) and IgG (#A7007, Beyotime, China) or a primary antibody at an ambient temperature of 4 °C with rotational shaking overnight. The next day, the beads were washed 6 times with RIPA buffer, and 60 µl of 1X loading buffer was added. Finally, the beads were boiled in hot water at 100 °C for 10 minutes. For western blotting, RIPA buffer containing protease inhibitor was added to the cells and placed on ice for 30 min. The lysate was aspirated into Eppendorf tubes and then centrifuged at 12000 × g for 15 min. The supernatant was removed from the new Eppendorf tubes, and 5X buffer was added. Next, the cell lysate was boiled in hot water (100 °C) for 10 minutes. Finally, the boiled protein lysates were electrophoresed on SDS-PAGE gels. Protein expression levels were measured by using ImageJ software (National Institutes of Health, USA).

### Real time RT-PCR analysis

Total RNA from cells was extracted using TRIzol reagent (#AG21102, Accurate Biotechnology, Hunan, China). RT-qPCR was performed using reverse transcription kits (#AG11728, Accurate Biotechnology, Hunan, China) and PCR kits (#AG11701, Accurate Biotechnology, Hunan, China) according to the manufacturer's instructions. All values were normalized to the corresponding GAPDH values, and the 2^-ΔΔ^Ct method was used to quantify the fold change.

### Chromatin immunoprecipitation (ChIP)-qPCR

The detail of ChIP-qPCR procedure was mentioned previously 9. The chromatin extraction kit (Abcam, ab117152, USA) and the ChIP Kit Magnetic - One Step (Abcam, ab156907, USA) were employed to performed the ChIP. The primers of genes were provided in the supplementary information.

### Cell proliferation assay

For Cell Counting Kit-8 (CCK-8) assay, CCK-8 reagent (#C0037, Beyotime) was added to each cell well and an absorbance of 450 nm was measured with a microplate reader.

All animal experiments were approved by the ethics committee of the Second Xiangya Hospital, Central South University (Approval number: 2021613). BALB/c nude mice (6 weeks old) were purchased from Vital River (Beijing, China). Cells were subcutaneously injected into the left side of the backs of the mice (1 × 107 cells per mouse). Tumor volume was calculated using the formula (L × W2)/2. Once the mice were euthanized, the tumors were excised and weighed.

### Tissue microarray and immunohistochemistry (IHC)

The tissue microarray slides (# U081ki01) were purchased from bioaitech, China. The tissue microarray specimens were immunostained with RNF26 and CBX7. The method of scoring of staining intensity was mentioned previously 10. Staining intensity was graded/scored in a blinded fashion: 1 = weak staining at 100 X magnification but little or no staining at 40X magnification; 2 = medium staining at 40X magnification; 3 = strong staining at 40X magnification 21. The degree of immunostaining was reviewed and scored by two independent pathologists who were blinded to the clinical details. A final staining index was calculated using the formula: staining intensity X percentage of positive cells.

### Statistical analysis

Data were expressed as means ± SD. Statistical significance was determined by one-way or two-way ANOVA using GRAPHPAD PRISM 5 software, San Diego, CA, USA. A statistical significance threshold of P-values < 0.05 was used.

Other methods were provided in the supplementary information.

## Results

### CBX7 is a protective factor in renal cell carcinoma

**Figure 1:**
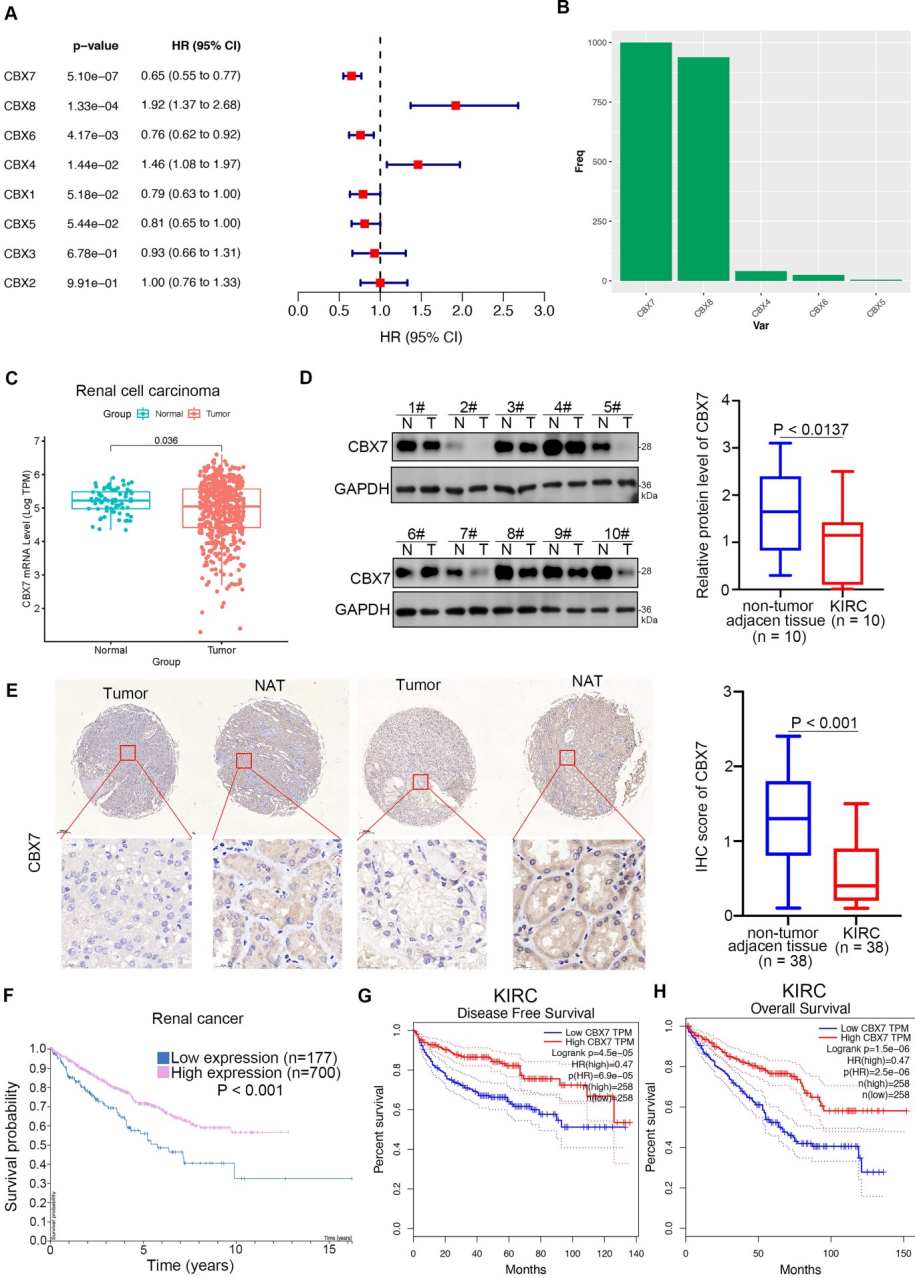
** CBX7 is a protective factor in renal cell carcinoma. A,** Univariate Cox regression analysis and multivariate Cox regression analysis adjusted for CBX1-8 in ccRCC. **B**, 10-fold cross-validation with 1000 replications for variable selection in the LASSO-COX-OS model by minimum criteria (the 1-SE criteria). **C**, Differential expression analyses of RNF26 between tumor and normal tissues in TCGA-KIRC dataset. **D**, The protein expression levels of CBX7 in the adjacent non-tumor renal tissues (n = 10) and ccRCC cancer tissues (n = 10) were analyzed by the western blot. The protein levels of CBX7 were quantified by the image J software. P values as indicated in panel D. **E**, IHC analysis of the tissue microarray by staining the CBX7 antibody. The typical image and expression level of CBX7 in the non-tumor renal tissue and renal cancer tissue were shown. P values as indicated. **F**, The CBX7 related overall survival in ccRCC analyzed by the Human protein atlas. **G and H**, The CBX7 related disease free survival and overall survival in ccRCC analyzed by the Human protein atlas.

Since the cancer-related role of the CBX family in renal cell carcinoma is unknown, we first performed bioinformatic analysis and found that CBX7 and CBX6 functioned as protective factors with hazard ratios (HRs) less than 1 but CBX8 and CBX4 functioned as risk factors with HRs greater than 1 in renal cell carcinoma (Fig. 1A). Least absolute shrinkage and selection operator (LASSO)-Cox regression analysis with 1000 replications for these eight CBX family members in the TCGA-KIRC dataset further showed that CBX7 might be a key gene related to overall survival (OS) in renal cell carcinoma (Fig. 1B). Analysis of the TCGA-KIRC dataset also revealed that CBX7 was downregulated in renal cancer tissues compared with nontumor renal tissues (Fig. 1C). This trend was also observed in bladder cancer, prostate cancer and other types of cancer (Supplementary Fig. 1A). Similarly, western blot analysis of the protein levels of CBX7 in patient samples with clear renal cell carcinoma from our hospital indicated that CBX7 was decreased in cancer tissues compared to adjacent nontumor (NAT) renal tissues (Fig. 1D). Moreover, the IHC staining assay of the renal cancer tissue microarray with the anti-CBX7 antibody showed that the protein levels of CBX7 in nontumor tissues were greater than those in renal cancer tissues (Fig. 1E). In addition, we demonstrated that high expression of CBX7 was closely correlated with favorable prognosis in RCC by using the Human Protein Atlas and the GEPIA web tool (Fig. 1F-H). Thus, our data suggest that CBX7 is downregulated in ccRCC and may be a predictor of favorable prognosis in ccRCC.

### CBX7 inhibits the progression of renal cancer

**Figure 2:**
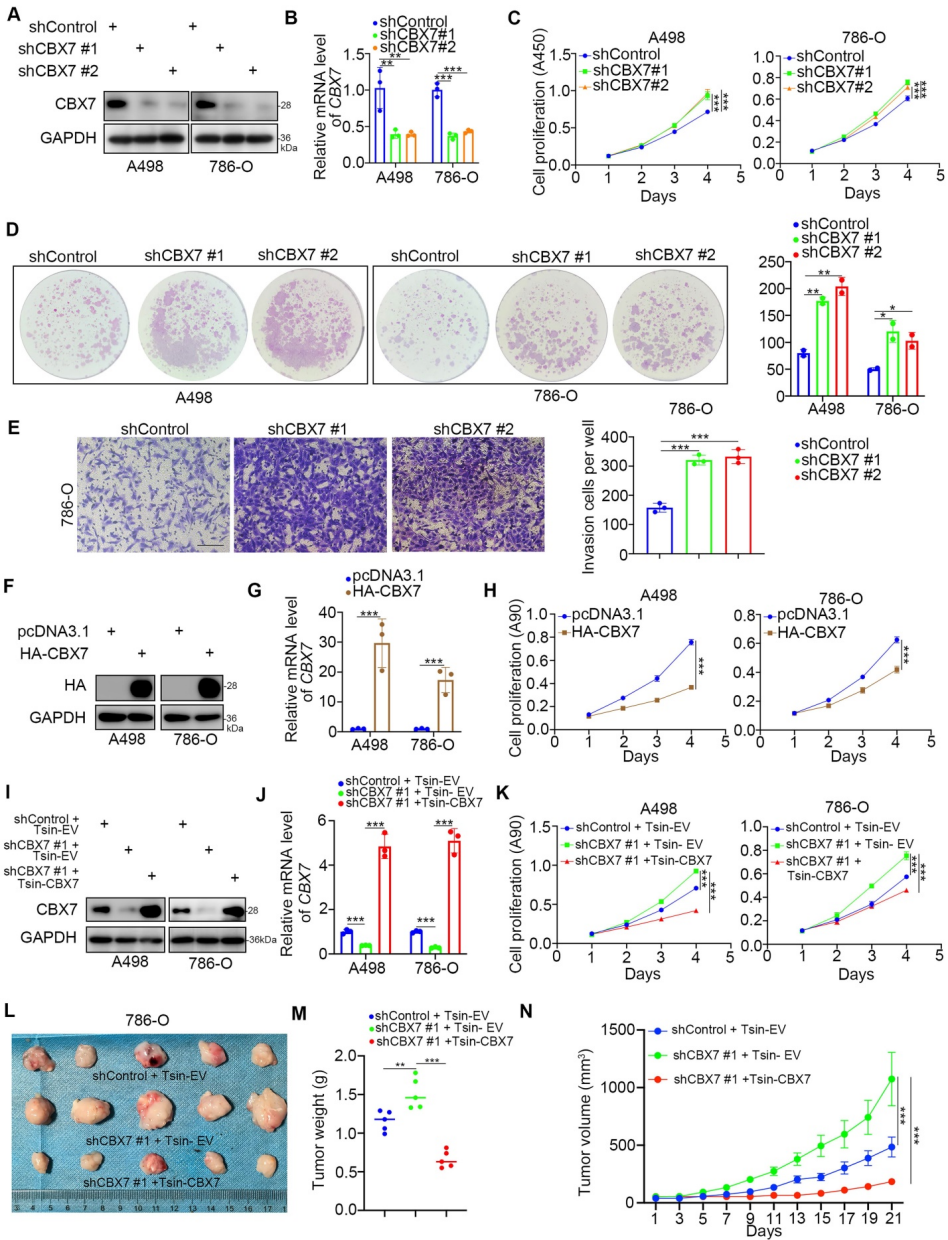
** CBX7 inhibits the progression of renal cancer. A-E,** A498 and 786-O cells were infected with shControl, shCBX7 #1, or shCBX7#2 for 72 h. Cells were collected for Western blot analysis (A), RT-qPCR analysis (B), CCK-8 assay (C), colony formation assay (D) and transwell assay (E). Data presents as mean ± SD with 3 replicates. *, P < 0.05; **, P < 0.01; ***, P < 0.001. **F-H**, A498 and 786-O cells were transfected with pcDNA3.1 or HA-CBX7 for 24 h. Cells were Cells were collected for Western blot analysis (F), RT-qPCR analysis (G) and CCK-8 assay (H). Data presents as mean ± SD with 3 replicates. ***, P < 0.001. **I-K**, A498 and 786-O cells were infected with indicated constructs for 72 h. Cells were harvested for Western blot analysis (I), RT-qPCR analysis (J) and CCK-8 assay (K). Data presents as mean ± SD with 3 replicates. ***, P < 0.001. **L-N**, 786-O cells were infected with indicated constructs for 72 h. After puromycin selection, cells were subcutaneously injected into the nude mice. The image of xenografts was shown in the panel I. The tumor mass was shown in the panel M. The tumor growth curve was shown in the panel N. Data presents as mean ± SD with 5 replicates. **, P < 0.01; ***, P < 0.001.

Given that the low expression of CBX7 indicated a worse prognosis of ccRCC, we then investigated the biological role of CBX7 in ccRCC cells. Intriguingly, the single-cell sequence of RCC, acute lymphoblastic leukemia, and ovarian cancer indicated that CBX7 was negatively correlated with angiogenesis, proliferation and invasion (Supplementary Fig. 1B-D). To further test the role of CBX7 in renal cancer cells, CBX7 was knocked down by using gene-specific shRNAs (Fig. 2A, B). CCK-8 and colony formation assays showed that silencing CBX7 enhanced the growth ability of renal cancer cells (Fig. 2C, D). We also demonstrated that knockdown of CBX7 promoted renal cancer cell invasion *in vitro* (Fig. 2E). In contrast, elevation of the CBX7 protein and mRNA levels led to reduced proliferation of renal cancer cells (Fig. 2F-H). Moreover, Tsin-CBX7 was transduced into renal cancer cells to rescue CBX7 expression (Fig. 2I, J). The CCK-8 assay and xenograft assay demonstrated that rescue of CBX7 expression overcame the growth-promoting effect induced by CBX7 downregulation (Fig. 2K-N). Together, these findings briefly show that CBX7 inhibits the aggressive behavior of renal cancer cells.

### CBX7 blocks ccRCC tumorigenesis by inhibiting the TNF pathway

**Figure 3:**
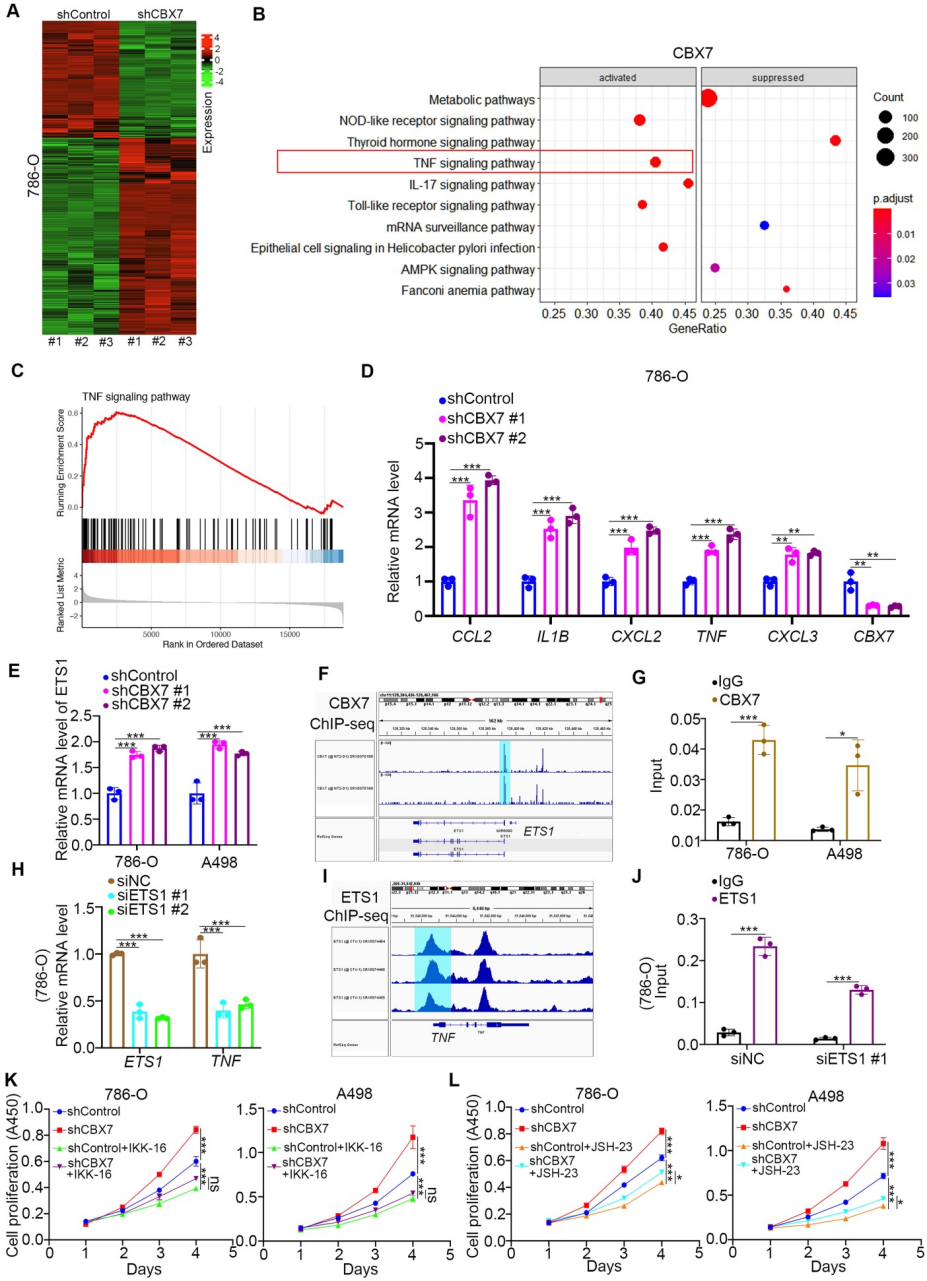
** CBX7 blocks ccRCC tumorigenesis by inhibiting the TNF pathway. A,** 786-O cells were infected with shControl or shCBX7 for 72 h. Cells were subjected to RNA-seq analysis. **B**, KEGG pathway enrichment analysis indicated that the pathways were changed after knockdown of CBX7 in 786-O cells. **C**, GSEA analysis showed TNF pathway was activated by knocking down CBX7 in 786-O cells. **D**, 786-O cells were infected with shControl or shCBX7 for 72 h. Cells were collected for RT-qPCR analysis. Data presents as mean ± SD with 3 replicates. **, P < 0.01; ***, P < 0.001. **E**, 786-O and A498 cells were infected with shControl or shCBX7 for 72 h. Cells were collected for RT-qPCR analysis. Data presents as mean ± SD with 3 replicates. ***, P < 0.001. **F**, the ChIP-seq of CBX7 showed the binding peak of CBX7 in the promoter region of ETS1. **G**, 786-O and A498 cells were subjected to ChIP-qPCR analysis by using the IgG and CBX7 antibodies. **H**, 786-O and A498 cells were transfected with siNC or siETS1 for 48 h. Data presents as mean ± SD with 3 replicates. ***, P < 0.001. **I**, the ChIP-seq of ETS1 showed the binding peak of ETS1 in the promoter region of TNF. **J**, 786-O cells were transfected with siNC or siETS1 for 48 h. Cells were harvested for ChIP-qPCR analysis by using the IgG and ETS1 antibodies. **K**, 786-O and A498 cells were infected with shControl

To further explore the mechanism of the tumor suppressive effect of CBX7 in ccRCC, RNA-seq was performed after knockdown of CBX7 (Fig. 3A). KEGG pathway enrichment analysis showed that CBX7 silencing activated the TNF pathway in 786-O cells (Fig. 3B). Gene set enrichment analysis (GSEA) also showed that knockdown of CBX7 was involved in the activation of the TNF signaling pathway (Fig. 3C). In addition, we demonstrated that CBX7 knockdown increased the expression of TNF pathway downstream genes, such as C-C motif chemokine ligand 2 (CCL2), interleukin 1 beta (IL1B), C-X-C motif chemokine ligand 2 (CXCL2), TNF, and CXCL3 (Fig. 3D).

As TNF is the stimulator of the TNF pathway, we performed ChIP-seq of CBX7 to determine whether CBX7 directly regulates the expression of TNF (Supplementary Fig. 2A). However, there was no binding peak of CBX7 in the promoter region of TNF (Supplementary Fig. 2A). Thus, we intended to find a mediator of CBX7-induced TNF downregulation in ccRCC. By combining the RNA-seq data and ChIP-seq dataset, we identified five candidates, including ETS proto-oncogene 1 (ETS1), serum response factor (SRF), lysine demethylase 2B (KDM2B), cyclin dependent kinase 8 (CDK8) and core-binding factor subunit beta (CBFB) (Supplementary Fig. 2B). ETS1 was the most changed candidate after CBX7 silencing, and this phenomenon was observed in renal cancer cells (Fig. 3E, Supplementary Fig. 2C). We also showed that overexpression of CBX7 decreased ETS1 expression in renal cancer cells (Supplementary Fig. 2D). Additionally, ChIP-seq and subsequent ChIP-qPCR assays showed that CBX7 directly bound to the promoter region of ETS1 (Fig. 3F, G). Then, we demonstrated that knockdown of ETS1 decreased TNF expression and that ETS1 directly bound to the promoter region of TNF in 786-O cells (Fig. 3H-J). Since the TNF signaling pathway is responsible for the regulation of tumor growth via activating NF-kappa B pathway in renal cancer and other types of malignant tumor 11, 12, we tested whether CBX7 inhibited tumor proliferation through inactivation of the TNF pathway. We found that TNF pathway inhibitors treatment (IKK-16 and JSH-23) diminished the tumor growth-promoting effect induced by CBX7 knockdown in both 786-O and A498 cells (Fig. 3K, L).

### CBX7 contributes to regulating sensitivity to TKIs in ccRCC

**Figure 4:**
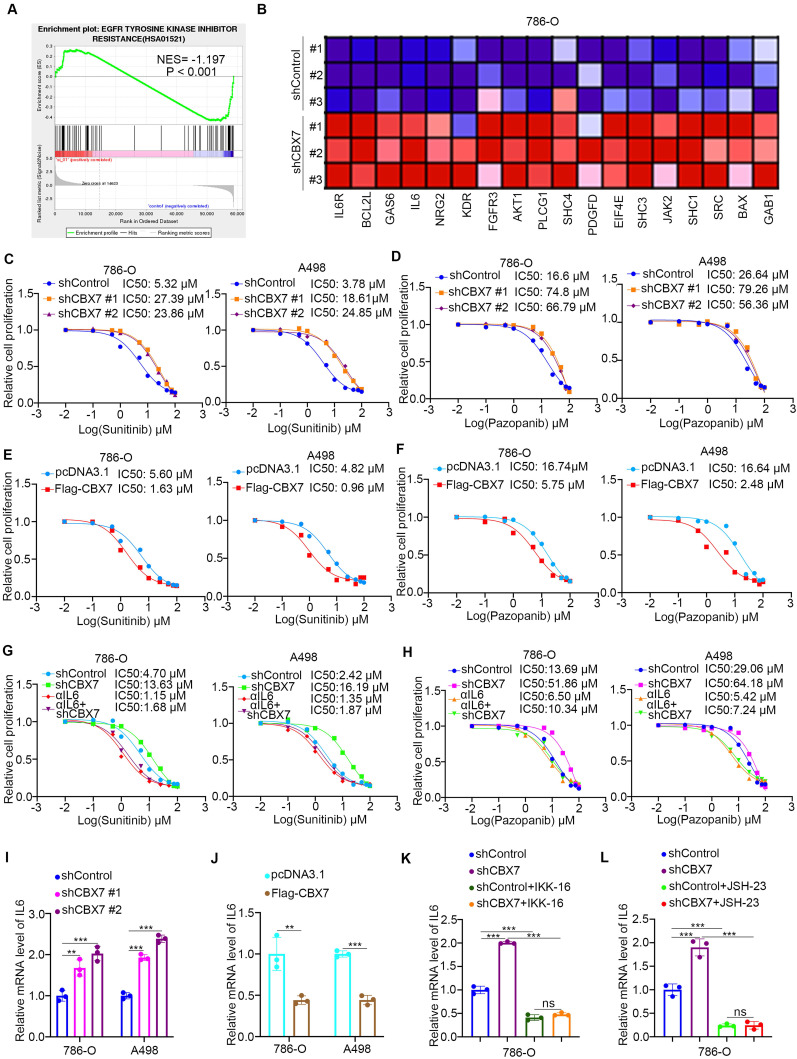
** CBX7 contributes to regulating sensitivity to TKIs in ccRCC. A,** GSEA analysis showed EGFR tyrosine kinase inhibitors resistance related pathway was activated by knocking down CBX7 in 786-O cells.** B**, the genes involved EGFR tyrosine kinase inhibitors resistance related pathway after knocking down CBX7 in 786-O cells. **C**, 786-O and A498 cells were infected with indicated shRNAs for 72 h. Cells were treated with a serial concentration of Sunitinib and harvested for CCK-8 assay. **D**, 786-O and A498 cells were infected with indicated shRNAs for 72 h. Cells were treated with a serial concentration of Pazopanib and harvested for CCK-8 assay. **E**, 786-O and A498 cells were transfected with indicated constructs for 24 h. Cells were treated with a serial concentration of Sunitinib and harvested for CCK-8 assay. **F**, 786-O and A498 cells were transfected with indicated constructs for 24 h. Cells were treated with a serial concentration of Pazopanib and harvested for CCK-8 assay. **G**, 786-O and A498 cells were infected with indicated shRNAs for 72 h. Cells were pretreated with or without IL6 for 6 h. Then, these cells were treated with a serial concentration of Sunitinib and harvested for CCK-8 assay. **H**, 786-O and A498 cells were transfected with indicated constructs for 24 h. Cells were pretreated with or without IL6 for 6 h. Then, these cells were treated with a serial concentration of Sunitinib and harvested for CCK-8 assay. **I**, 786-O and A498 cells were infected with indicated shRNAs for 72 h. Cells were harvested for RT-qPCR analysis. Data presents as mean ± SD with 3 replicates. **, P < 0.01; ***, P < 0.001. **J**, 786-O and A498 cells were transfected with indicated plasmids for 24 h. Cells were harvested for RT-qPCR analysis. Data presents as mean ± SD with 3 replicates. **, P < 0.01; ***, P < 0.001. **K**, 786-O cells were infected with indicated shRNAs for 72 h. Cells were treated with or without IKK-16 for another 24 h. Cells were harvested for RT-qPCR analysis. Data presents as mean ± SD with 3 replicates. ***, P < 0.001. **L**, 786-O cells were infected with indicated shRNAs for 72 h. Cells were treated with or without JSH-23 for another 24 h. Cells were harvested for RT-qPCR analysis. Data presents as mean ± SD with 3 replicates. ***, P < 0.001.

Tyrosine kinase inhibitors (TKIs) are the first-line therapy for ccRCC. Of note, gene set enrichment analysis (GSEA) of the RNA-seq data for CBX7 indicated that CBX7 might be involved in EGFR tyrosine kinase inhibitor resistance (Fig. 4A), and the levels of a series of genes related to modulating sensitivity to TKIs were found to be changed after CBX7 silencing (Fig. 4B). The subsequent cellular study showed that knockdown of CBX7 increased the IC50 values of sunitinib and pazopanib in both 786-O and A498 cells (Fig. 4C, D). In contrast, overexpression of CBX7 reduced the IC50 values of sunitinib and pazopanib in renal cancer cells (Fig. 4E, F). We found that interleukin 6 (IL6) was upregulated after CBX7 silencing, as shown in Figure 4B, and IL6 is a well-known factor responsible for the resistance of renal cancer cells to TKIs. It was not surprising that IL6 neutralizing antibodies attenuated the CBX7-induced sensitivity change of TKIs (Fig. 4G, H). Then, we explored the mechanism by which CBX7 regulates the expression of IL6 in renal cancer cells. Although knockdown/overexpression of CBX7 increased/decreased IL6 expression in ccRCC cells (Fig. 4I, J,Supplementary Fig. 2C,2D), ChIP-seq of CBX7 demonstrated that CBX7 did not directly bind to the promoter region of IL6 (Supplementary Fig. 2E). IL6 is the downstream gene of TNF signaling 13, and CBX7 contributes to the repression of TNF signaling in renal cancer cells (Figure 3). We showed that inhibitors of TNF signaling diminished the increase in IL6 after CBX7 knockdown in 786-O cells (Fig. 4K, L). These results showed that CBX7 regulated IL6 expression through TNF signaling pathway. Thus, we revealed that CBX7 regulated sensitivity to TKIs through the TNF/IL6 axis in ccRCC.

### RNF26 interacts with CBX7 and decreases CBX7 expression in ccRCC

**Figure 5:**
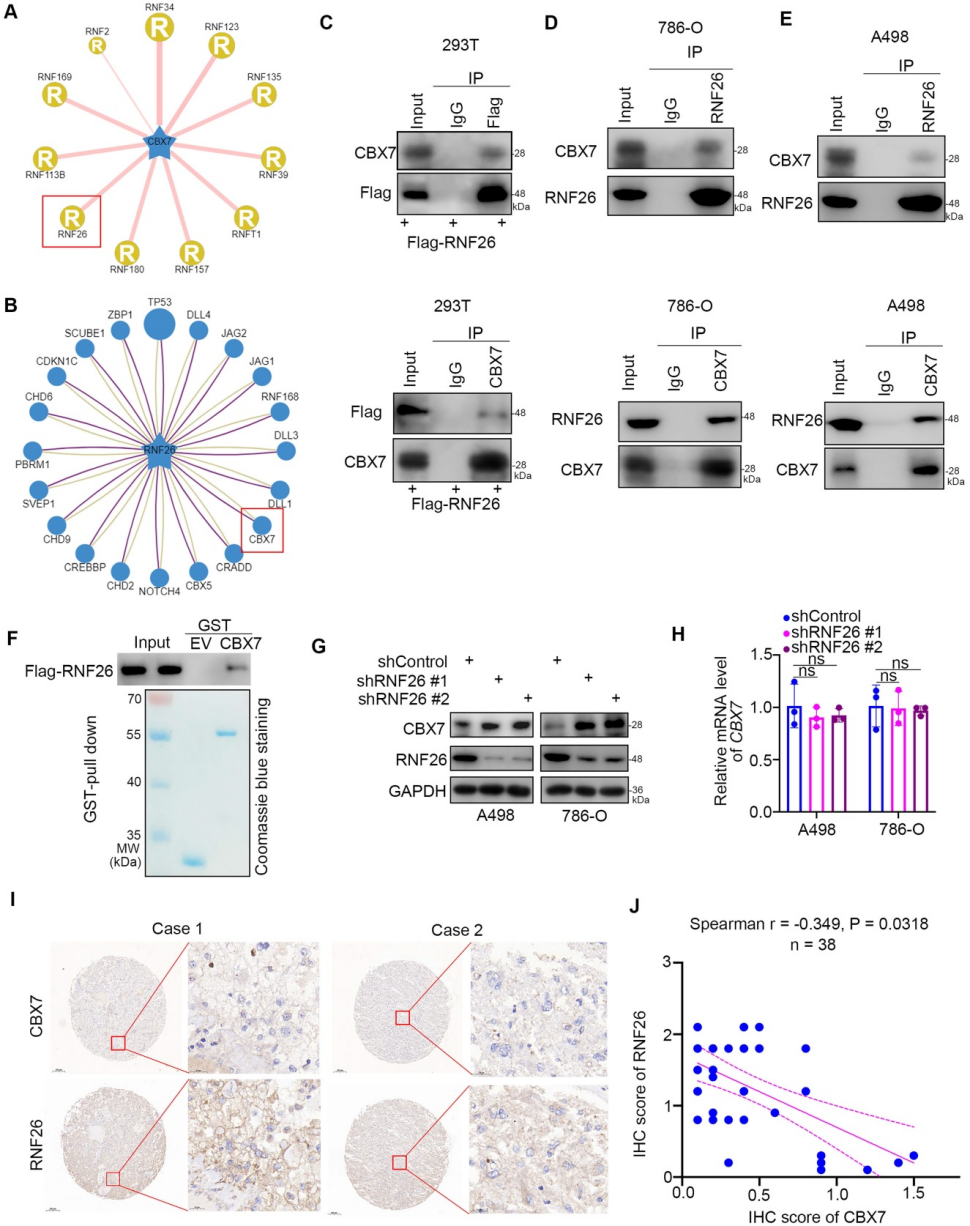
** RNF26 interacts with CBX7 and decreases CBX7 expression in ccRCC. A,** UbiBrowser showed substrate proteins that might interact with CBX7.** B,** UbiBrowser showed substrate proteins that might interact with RNF26.** C,** 293T cells were transfected with indicated plasmids. Cells were collected and immunoprecipitated with IgG and CBX7 or Flag antibodies. **D and E,** 786-O and A498 cells were harvested and immunoprecipitated with IgG and CBX7 or RNF26 antibodies. **F**, Western blot analysis for Flag-RNF26 in 786-O cells after GST or GST-CBX7 pulldown. The bottom panel shows the Coomassie blue staining of GST or GST-CBX7 protein input. **G and H**, A498 and 786-O cells were infected with indicated shRNAs for 72 h. Cells were harvested for Western blot (G) and RT-qPCR analysis. Data presents as mean ± SD with 3 replicates. Ns, not significant. **I and J**, Renal cancer tissue microarray was stained for CBX7 and RNF26. Representative images are shown in panel I. The correlation of CBX7 and RNF26 levels is shown in panel J; P-values are also shown in the figure.

Since downregulation of CBX7 is important for the progression of ccRCC, the regulatory mechanism of CBX7 in ccRCC is worthy of further study. Regulation of protein stability by ubiquitination is a major aspect of posttranslational modification. Protein-protein interaction (PPI) network analysis indicated that the E3 ligase RNF26 was closely associated with CBX7 in renal cancer (Fig. 5A, B). The coimmunoprecipitation assay showed that exogenously expressed CBX7 bound to RNF26 in 293T cells (Fig. 5C). Then, we found that endogenously expressed CBX7 interacted with RNF26 in both 786-O and A498 cells (Fig. 5D, 5E). Consistent with this result, the GST pulldown assay demonstrated that there was an interaction between RNF26 and CBX7 *in vitro* (Fig. 5F). We further evaluated the relationship between RNF26 and CBX7 in renal cancer cells after knocking down RNF26. We found that RNF26 silencing increased the protein levels of CBX7 and had no effect on the mRNA levels of CBX7 in renal cancer cells (Fig. 5G, H). Moreover, IHC staining of a renal cancer tissue microarray with anti-CBX7 and anti-RNF26 antibodies demonstrated that CBX7 expression was negatively correlated with RNF26 expression in the patient specimens (Spearman correlation r = -0.349, P = 0.0318) (Fig. 5I, J). Therefore, our results indicate that RNF26 is a binding partner of CBX7 and is involved in decreasing the protein level of CBX7 in RCC.

### RNF26 promotes the degradation of CBX7 in renal cancer cells

**Figure 6:**
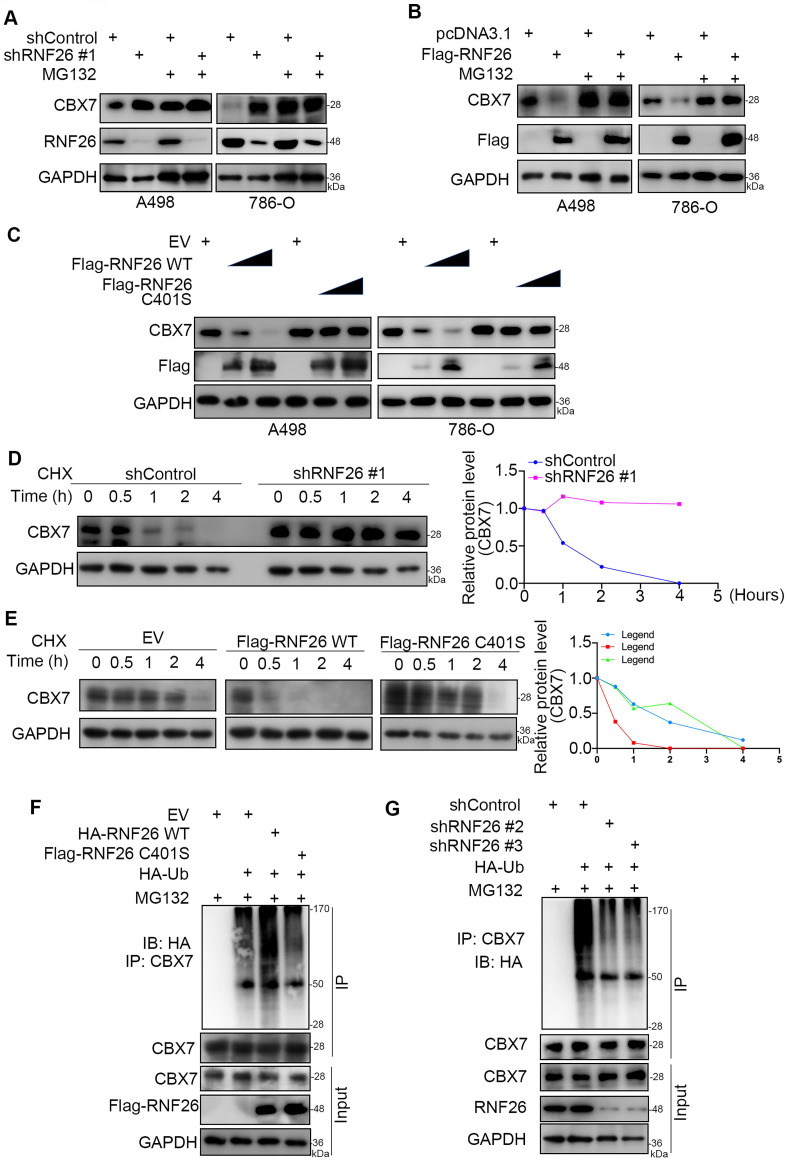
** RNF26 promotes the degradation of CBX7 in renal cancer cells. A,** A498 and 786-O cells were infected with indicated shRNAs for 72 h. After treated with or without MG132 for another 8 h, cells were harvested for Western blot analysis. **B**, A498 and 786-O cells were transfected with indicated plasmids for 24 h. After treated with or without MG132 for another 8 h, cells were harvested for Western blot analysis. **C**, A498 and 786-O cells were transfected with indicated plasmids for 24 h. Cells were harvested for Western blot analysis. **D**, 786-O cells were infected with the indicated shRNAs. After 72 h, cells were treated with cycloheximide (CHX), and cells were collected for Western blot analysis at different time points. **E**, 786-O cells were transfected with the indicated plasmids. After 24 h, cells were treated with CHX, and cells were collected for Western blot analysis at different time points. **F**, 786-O cells were transfected with the indicated plasmids. After 24 h, cells were collected for Western blot after treatment with MG132 for 8 h. **G**, 786-O cells were infected with the indicated shRNAs. After 72 h, cells were collected for Western blot after treatment with MG132 for 8 h.

To further explore the relationship between RNF26 and CBX7 in renal cancer cells, we treated renal cancer cells with or without a 26S proteasome inhibitor (MG132) under conditions of knockdown or overexpression of RNF26 (Fig. 6A, B). We showed that MG132 effectively blocked the upregulation or downregulation of CBX7 induced by knockdown or overexpression of RNF26 in A498 and 786-O cells (Fig. 6A, B). Moreover, we showed that overexpression of wild-type (WT) RNF26 but not the functionally dead mutant (C401S) reduced the protein level of CBX7 in A498 and 786-O cells 14 (Fig. 6C). In addition, we found that knockdown of RNF26 prolonged the protein half-life of CBX7 and deceased the polyubiquitination level of CBX7 in 786-O cells (Fig. 6d, g). However, overexpression of RNF26 WT but not the C401S mutant shortened the protein half-life of CBX7 and increased the polyubiquitination level of CBX7 in 786-O cells (Fig. 6e, f). Thus, our data indicated that RNF26 regulated the protein stability of CBX7 in renal cancer cells.

### CBX7 is a key mediator of RNF26-induced RCC progression

**Figure 7:**
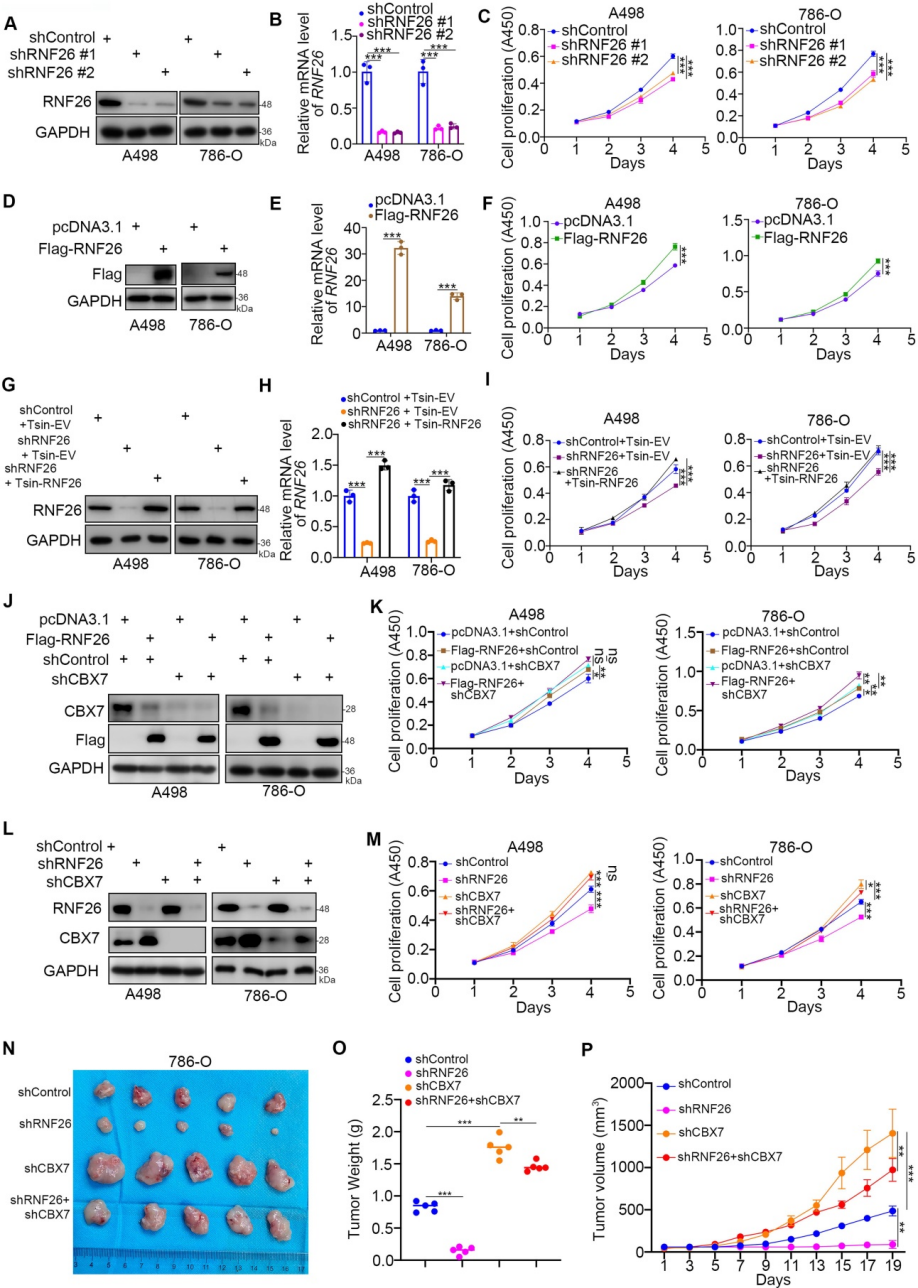
** CBX7 is a key mediator of RNF26-induced RCC progression. A-C,** A498 and 786-O cells were infected with indicated shRNAs for 72 h. Cells were collected for Western blot analysis (A), RT-qPCR (B) and CCK-8 assay (C). Data presents as mean ± SD with 3 replicates. ***, P < 0.001. **D-F**, A498 and 786-O cells were transfected with indicated plasmids for 24 h. Cells were collected for Western blot analysis (D), RT-qPCR (E) and CCK-8 assay (F). Data presents as mean ± SD with 3 replicates. ***, P < 0.001. **G-I**, A498 and 786-O cells were infected with indicated constructs for 72 h. Cells were collected for Western blot analysis (G), RT-qPCR (H) and CCK-8 assay (I). Data presents as mean ± SD with 3 replicates. ***, P < 0.001.** J and K**, A498 and 786-O cells were infected with indicated constructs for 72 h. Cells were collected for Western blot analysis (J) and CCK-8 assay (K). Data presents as mean ± SD with 3 replicates. Ns, not significant; *, P < 0.05; **, P < 0.01; ***, P < 0.001. **L and M**, A498 and 786-O cells were infected with indicated shRNAs for 72 h. Cells were collected for Western blot analysis (L) and CCK-8 assay (M). Data presents as mean ± SD with 3 replicates. Ns, not significant; *, P < 0.05; ***, P < 0.001. **N-P**, 786-O cells were infected with indicated constructs for 72 h. After puromycin selection, cells were subcutaneously injected into the nude mice. The image of xenografts was shown in the panel N. The tumor mass was shown in the panel O. The tumor growth curve was shown in the panel P. Data presents as mean ± SD with 5 replicates. **, P < 0.01; ***, P < 0.001.

Next, we evaluated the cancer-related role of RNF26 in RCC. It was not surprising that knockdown of RNF26 decreased the proliferation ability of renal cancer cells (Fig. 7A-C). In contrast, overexpression of RNF26 promoted renal cancer cell growth (Fig. 7D-F). In addition, rescue of RNF26 expression reversed the growth-suppressive effect induced by RNF26 silencing (Fig. 7G-I). Notably, compared with overexpression of RNF26 alone, overexpression of RNF26 in CBX7 knockdown cells did not further increase cancer cell growth (Fig. 7J, K). In addition, we also showed that combined knockdown of CBX7 and RNF26 attenuated the tumor growth inhibition effect induced by knockdown of RNF26 alone in renal cancer cells and a subcutaneous xenograft model (Fig. 7L-P). Together, our data suggest that RNF26 might promote renal cell proliferation via CBX7.

## Discussion

Studies have reported that different CBX family members perform distinct functions in cancer. Recently, Zhu, et al performed comprehensive analysis of the expression and prognosis value of CBX1-8 in ccRCC 15. They demonstrated that CBX3 and CBX4 were up-regulated in the ccRCC tissues and associated with the poor prognosis, but CBX1, CBX5, CBX6, and CBX7 were down-regulated in the ccRCC tissues and correlated with the favorable prognosis 15. In this study, we performed bioinformatic analysis to show that CBX7 and CBX6 functioned as protective factors with hazard ratios (HRs) less than 1 but CBX8 and CBX4 functioned as risk factors with HRs greater than 1 in renal cell carcinoma (Fig. 1A). The clinicopathological roles of CBX4 and CBX7 were consistent in these two studies with opposite roles in ccRCC, respectively. Here, we focused on studying the cancer related role of CBX7 in ccRCC. CBX7 expression is lost in a variety of cancers, such as urothelial carcinoma 16, colorectal cancer 17, breast cancer 18, pancreatic cancer 19 and lung cancer 20. In urothelial carcinoma, the expression of CBX7 showed a close correlation with high tumor grade 16. In addition, a clear correlation between downregulation of CBX7 and poor prognosis has been found in many types of cancers 21. Consistent with previous findings, our data indicated that the CBX7 level was decreased in ccRCC and was a protective factor for ccRCC prognosis. In addition, the tumor-associated role of CBX7 has been studied. It has been reported that CBX7 hinders HDAC2 activity to promote CDH1 expression and block epithelial-mesenchymal transition in thyroid, pancreatic and cervical cancer cells 19, 22-24. In addition, CBX7 has been found to suppress pancreatic cancer progression by inhibiting AKT signaling 25 and to inhibit tumorigenicity in breast cancer through the Wnt/β-catenin pathway 26. However, CBX7 also acts as an oncogenic protein in gastric cancer by upregulating p16 27. Thus, the role of CBX7 in cancer is controversial. Here, we demonstrated that CBX7 inactivated the TNF signaling pathway through downregulation of ETS1 expression in ccRCC cells, which was reported to transcriptionally increase the expression of TNF 28. Several pathways contribute to the progression of ccRCC, such as Hypoxia signaling pathway [29]and PI3K-AKT signaling pathway 30. Of them, TNF alpha mediated NF-kappa B signaling activation also plays a key role in promoting the tumor growth in ccRCC 11. It has been reported that TNF alpha induced eotaxin-1 expression to enhance the progression of renal cancer cells through CCR3 11. Recently, Du et al have demonstrated that activation of the NF-kappa B pathway increased the ccRCC tumor growth capability through upregulating stathmin 31. Meanwhile, the absence of *von Hippel Lindau gene* (VHL) gene enhances the activity of NF-kappa B, which subsequently leads to drug resistance and epithelial-mesenchymal-transition of RCC 32. Here, we showed that CBX7 blocked tumor proliferation through inactivation of the TNF pathway and regulated sensitivity to TKIs via the TNFα/IL6 axis. While, the TNFα/NF-kappa B signaling was not the only pathway for CBX7 to inhibit the tumor growth in ccRCC, the underlying mechanism by which CBX7 modulates the cancer progression needs to be performed in the furture.

CBX7 loss is a common feature in many cancers, including renal cancer. The underlying mechanism needs in-depth study. HMGA1 has been found to directly bind to the promoter of CBX7 and transcriptionally repress CBX7 expression 18. In addition, several microRNAs, such as miR-421, miR-181b, miR182 and miR-183, have been reported to suppress CBX7 expression 21, 33. Separate from the transcriptional regulation of CBX7, we focused on the posttranscriptional modification of CBX7 in renal cancers. We demonstrated that RNF26 interacted with CBX7 and promoted CBX7 degradation in renal cancer cells, which provides novel insight into the low expression of CBX7 in cancers. Of note, the PPI network analysis suggested that CBX7 might bind with other E3 ligases. Thus, the regulatory mechanism of CBX7 degradation needs further exploration.

RNF26 belongs to the RING finger protein with a C-terminal RING finger domain 34. RNF26 promotes K11-linked polyubiquitination of MITA and degradation of IRF3 to trigger type I interferon 14. Meanwhile, the endoplasmic reticulum (ER)-located RNF26 induces the ubiquitination of the scaffold p62/sequestosome 1 and regulates of the endocytic and exocytic systems 35. The dysregulation of RNF26 in cancer is rarely reported. We previously showed that RNF26 was abnormally upregulated in bladder cancer and promoted the tumor growth through destabilizing p57 36. Here, we demonstrated that CBX7 was another substrate of RNF26 for degradation in renal cancer cells. And RNF26 degraded CBX7 to promote the renal cancer cells proliferation. We believed that more substrates of RNF26 will be identified in the further to explain the role of RNF26 in malignant tumor.

**Figure 8:**
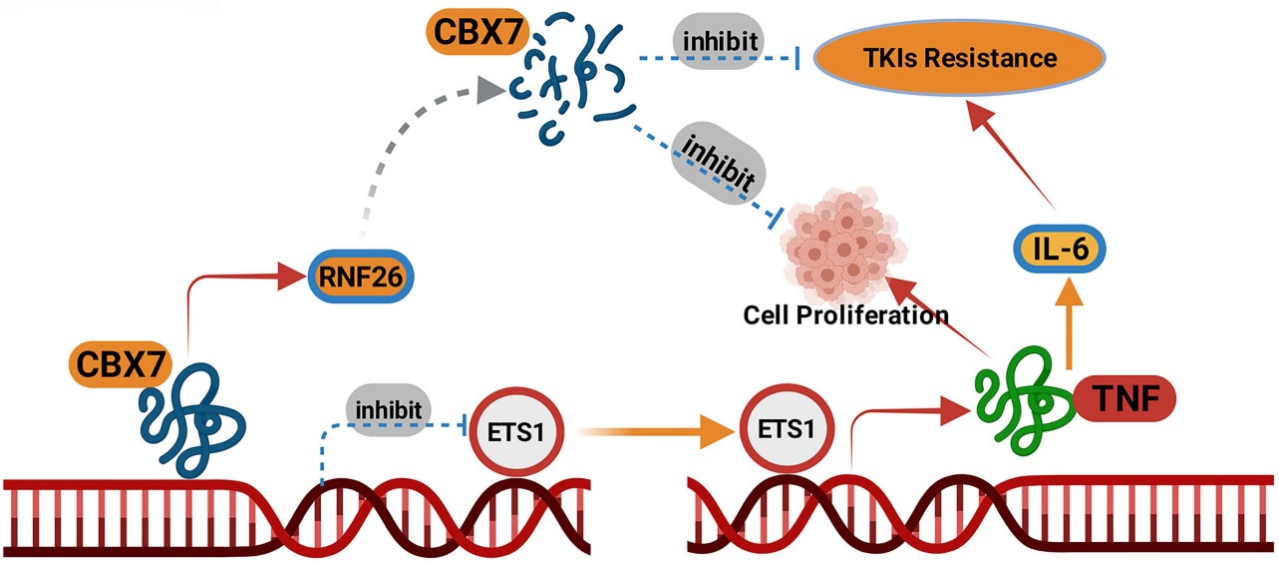
A model depicting that CBX7was degraded by RNF26. The downregulated CBX7 inhibited the ETS1 expression and subsequently inactivated TNF pathway to block the tumor growth and overcome the TKIs resistance in ccRCC.

Collectively, our results demonstrated that CBX7 was downregulated in ccRCC tissues and associated with favorable prognosis in patients with ccRCC. Then, we showed that CBX7 inactivated the TNF signaling pathway to inhibit tumor proliferation and enhanced the sensitivity of ccRCC cells to TKIs. Moreover, we found that CBX7 was a bona fide substrate of RNF26. RNF26 promoted the degradation of CBX7 and enhanced ccRCC tumor growth (Fig. 8). Therefore, our results revealed a novel RNF26/CBX7 axis that modulates the TNF signaling pathway in ccRCC.

## Supplementary Material

Supplementary materials and methods, figures and tables.

## Abbreviations

TCGA: The Cancer Genome Atlas
ANOVA: one-way analysis of variance
KIRC: Kidney Renal Clear Cell Carcinoma
GEPIA: Gene Expression Profiling Interactive Analysis
RNF26: ring finger protein 26
TKI: tyrosine kinase inhibitors
CBX7: Chromobox protein homolog 7
ChIP: Chromatin immunoprecipitation
IHC: immunohistochemistry
ccRCC: Clear cell renal cell carcinoma
ETS1: ETS proto-oncogene 1
SRF: serum response factor
KDM2B: lysine demethylase 2B
CDK8: cyclin dependent kinase 8
CBFB: core-binding factor subunit beta
RNF: ring finger protein
TNF: tumor necrosis factor
HDAC1: histone deacetylase 1
KLF6: Kruppel like factor 6
CCL2: C-C motif chemokine ligand 2
IL1B: interleukin 1 beta
CXCL2: C-X-C motif chemokine ligand 2
IL6: interleukin 6
