# Supplementary material for: The RNF26/CBX7 axis modulates the TNF pathway to promote cell proliferation and regulate sensitivity to TKIs in ccRCC
Source: Int J Biol Sci. 2022 Feb 28;18(5):2132–45. doi: 10.7150/ijbs.69325 (PMC8935234; doi:10.7150/ijbs.69325)
Supplement: Supplementary file 1 — Supplementary materials and methods, figures and tables. [file ijbsv18p2132s1.pdf]

# The RNF26/CBX7 axis modulates the TNF pathway to promote the proliferation and regulate the sensitivity of TKIs in ccRCC

Wentao Liu, Haohui Wang, Chengzhu Jian, Wei Li, Kun Ye, Jiannan Ren , Liang Zhu, Yinhuai Wang, Xin Jin, Lu Yi

Supplementary figure 1

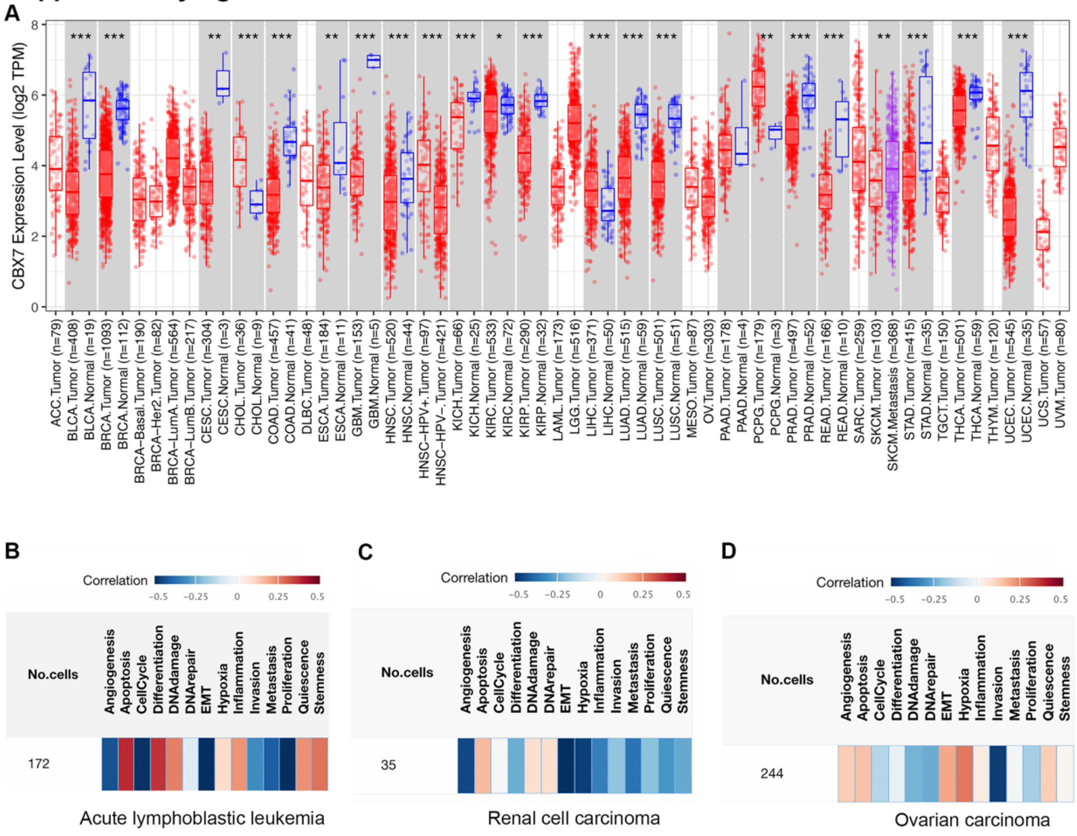


**Supplementary figure 1. A,** the expression level of CBX7 in the different types of cancer tissues and adjacent normal tissues. **B-D,** the single cell RNA-seq analysis indicated the biological role of CBX7 on Acute lymphoblastic leukemia (B), Renal cell carcinoma (C), Ovarian carcinoma (D).

## Supplementary figure 2

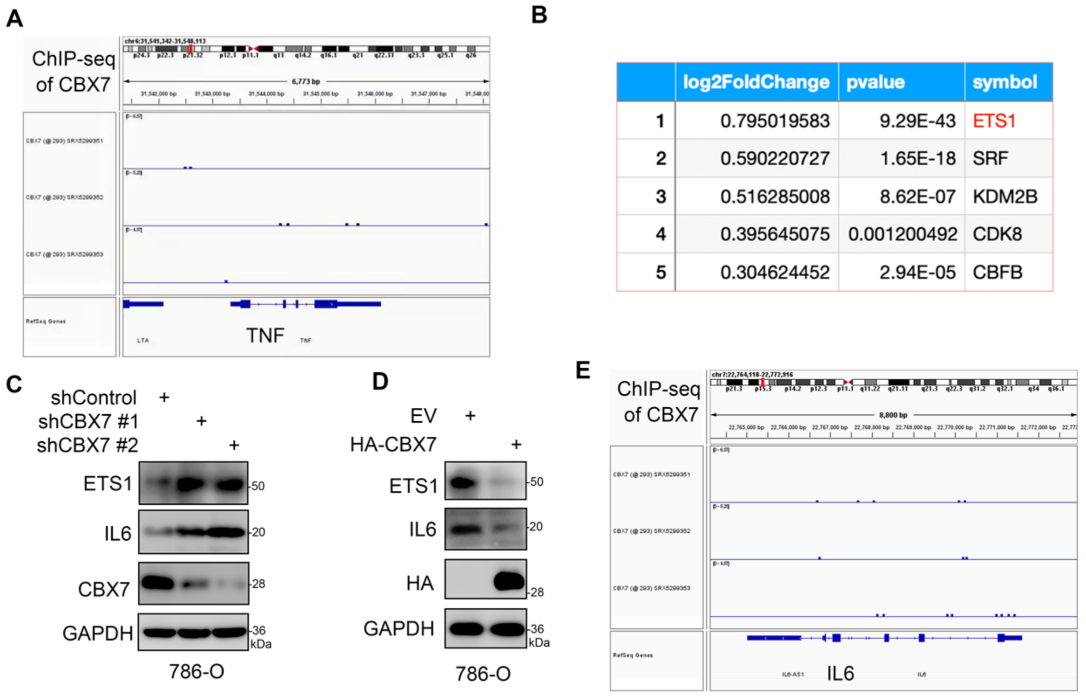


**Supplementary figure 2. A,** the ChIP-seq of CBX7 on the promoter of TNF. **B**, the potential genes regulated by the CBX7 had the binding peaks on the TNF. **C**, 786-O cells were infected with indicated shRNAs for 72 h. Cells were harvested for Western blot analysis. **D**, 786-O cells were transfected with indicated plasmids for 24 h. Cells were harvested for Western blot analysis. **E,** the ChIP-seq of CBX7 on the promoter of IL-6

**Supplementary Material and Methods**

**Public datasets for data mining and bioinformatics analysis**

**Survival analysis**

KIRC patients were divided into two groups according to the median expression level of the key gene. The differences in RFS and OS between the high and low expression groups were evaluated by the Kaplan–Meier method, followed by a log-rank test.

**GSEA for the key gene**

GSEA: KIRC patients were first divided into two groups according to the median expression level of the key gene. Then, differential expression analysis was applied between the high and low expression groups. Input genes for GSEA were sorted by their logFC values. Signaling pathways activated or suppressed by the key gene were decided by the normalized enrichment score (NES) value derived from GSEA.

ssGSEA: ssGSEA was used to calculate separate enrichment scores for each pairing of a KIRC sample and KEGG gene set. The ssGSEA score was further rescaled by min-max normalization method. Correlation analysis was performed between expression values of key gene and NES of signaling pathways.

**Upstream targets of the key gene**

Upstream targets of the key gene were determined by ChIP-seq and correlation analysis. Binding site in the targeted gene promoter of the key protein or methylation were obtained from ChIP-Atlas database (https://chip-atlas.org/). Correlation analysis between gene/protein and targeted genes was applied to further validate the ChIP-seq results.

**Predicted substrates for E3 ligase**

UbiBrowser (http://ubibrowser.ncpsb.org.cn/) was used to query potential substrates of a given E3 Ligase.

**Statistical analysis and visualization**

Microsoft R Open v4.0.2 was used for data mining, bioinformatics analysis and visualization in transcriptomics data. IGV v2.9.0 was used for analysis and visualization of ChIP-seq data.

**Table S1. The shRNA and siRNA sequences.**

| | |
|---|---|
| ShRNF26 #1 | 5'-CCGGCACCGCGGAGTCTTGCTTTCATTGCTTCAAGAGAGCAATGAAAGCAAGACTCCGCTTTTTG-3' |
| ShRNF26 #2 | 5'-CCGGCACCGCCGTGGTCCGGTTCACATGTTCAAGAGAACATGTGAACCGGACCACGGCTTTTTG-3' |
| ShRNF26 #3 | 5'-CCGGCACCGGAGCCTCCAGGTGGGCAAGATTCAAGAGATCTTGCCCACCTGGAGGCTCCTTTTTT-3' |
| ShCBX7 #1 | 5'-CACCGCAAGGAGAAGCTCTGCTTTTCAAGAGAAAGCAGAGCTTCTCCTTGCTTTTTT-3' |
| ShCBX7 #2 | 5'-CACCACTCCATCACCGTCACCTTTTCAAGAGAAAGGTGACGGTGATGGAGTTTTTT-3' |
| siETS1 #1 | 5'-AAGCATTAAAAGCTACTTTCAGT-3' |
| siETS1 #2 | 5'-GTGTAGACTTCCAGAAGTTCTGT-3' |

**Table S2. The primer sequences for RT-qPCR.**

| Gene (Human) | Forward primer (5′ - 3′) | Reverse primer (5′ - 3′) |
|---|---|---|
| GAPDH | ATGACAATGAATACGGCTACAGCA | GCAGCGAACTTTATTGATGGTATT |

| | | |
|---|---|---|
| RNF26 | TCGGCACTCAGAACCTCTTT | GCCAGTGAGATTGACAAGCA |
| CBX7 | GAAGCTCTGCTTCTCCCTGA | CAGGTACTTGTGGGCCTTTC |
| CCL2 | CCCCAGTCACCTGCTGTTAT | TGGAATCCTGAACCCACTTC |
| IL1B | GGGCCTCAAGGAAAAGAATC | TTCTGCTTGAGAGGTGCTGA |
| CXCL2 | GCAGGGAATTCACCTCAAGA | GGATTTGCCATTTTTCAGCA |
| TNF | CAGAGGGCCTGTACCTCATC | GGAAGACCCCTCCCAGATAG |
| CXCL3 | GCAGGGAATTCACCTCAAGA | GGTGCTCCCCTTGTTCAGTA |
| ETS1 | TGGAGTCAACCCAGCCTATC | TCTGCAAGGTGTCTGTCTGG |
| IL6 | TACCCCCAGGAGAAGATTCC | TTTTCTGCCAGTGCCTCTTT |

**Table S3. The primer sequences for ChIP-qPCR.**

| Gene | Forward primer (5′ - 3′) | Reverse primer (5′ - 3′) |
|---|---|---|
| ETS1 primer (CBX7) | CTCCTCCTTCTCCTCCTCGT | AGCCGACTCTCACCATCATC |
| TNF primer (ETS1) | CCCTGAAAACAACCCTCAGA | AAGAGGCTGAGGAACAAGCA |
